# Fecal Immunochemical Test Screening and Risk of Colorectal Cancer Death

**DOI:** 10.1001/jamanetworkopen.2024.23671

**Published:** 2024-07-19

**Authors:** Chyke A. Doubeni, Douglas A. Corley, Christopher D. Jensen, Theodore R. Levin, Nirupa R. Ghai, Kimberly Cannavale, Wei K. Zhao, Kevin Selby, Skye Buckner-Petty, Ann G. Zauber, Robert H. Fletcher, Noel S. Weiss, Joanne E. Schottinger

**Affiliations:** 1 Department of Family and Community Medicine, The Ohio State University College of Medicine, Columbus; 2 Center for Health Equity, The Ohio State University Wexner Medical Center, Columbus; 3 Division of Research, Kaiser Permanente Northern California, Oakland; 4 Department of Health Systems Science, Kaiser Permanente Bernard Tyson School of Medicine, Pasadena, California.; 5 Department of Quality and Systems of Care, Kaiser Permanente Southern California, Pasadena; 6 Department of Research & Evaluation, Kaiser Permanente Southern California, Pasadena; 7 Center for Primary Care and Public Health (Unisanté), University of Lausanne, Lausanne, Switzerland; 8 Mayo Clinic, Phoenix, Arizona; 9 Department of Epidemiology and Biostatistics, Memorial Sloan Kettering Cancer Center, New York, New York; 10 Department of Population Medicine, Harvard Medical School, Boston, Massachusetts; 11 Department of Epidemiology, University of Washington, Seattle

## Abstract

**Importance:**

The fecal immunochemical test (FIT) is widely used for colorectal cancer (CRC) screening, but evidence of its effectiveness is limited.

**Objective:**

To evaluate whether FIT screening is associated with a lower risk of dying from CRC overall, according to cancer location, and within demographic groups.

**Design, Setting, and Participants:**

This nested case-control study in a cohort of screening-eligible people was conducted in 2 large, integrated health systems of racially, ethnically, and socioeconomically diverse members with long-term programs of mailed FIT screening outreach. Eligible participants included people aged 52 to 85 years who died from colorectal adenocarcinoma between 2011 and 2017 (cases); cases were matched in a 1:8 ratio based on age, sex, health-plan membership duration, and geographic area to randomly selected persons who were alive and CRC-free on case’s diagnosis date (controls). Data analysis was conducted from January 2002 to December 2017.

**Exposures:**

Completing 1 or more FIT screenings in the 5-year period prior to the CRC diagnosis date among cases or the corresponding date among controls; in secondary analyses, 2- to 10-year intervals were evaluated.

**Main Outcomes and Measures:**

The primary study outcome was CRC death overall and by tumor location. Secondary analyses were performed to assess CRC death by race and ethnicity.

**Results:**

From a cohort of 2 127 128 people, a total of 10 711 participants (3529 aged 60-69 years [32.9%]; 5587 male [52.1%] and 5124 female [47.8%]; 1254 non-Hispanic Asian [11.7%]; 973 non-Hispanic Black [9.1%]; 1929 Hispanic or Latino [18.0%]; 6345 non-Hispanic White [59.2%]) was identified, including 1103 cases and 9608 controls. Among controls during the 10-year period prior to the reference date, 6101 (63.5%) completed 1 or more FITs with a cumulative 12.6% positivity rate (768 controls), of whom 610 (79.4%) had a colonoscopy within 1 year. During the 5-year period, 494 cases (44.8%) and 5345 controls (55.6%) completed 1 or more FITs. In regression analysis, completing 1 or more FIT screening was associated with a 33% lower risk of death from CRC (adjusted odds ratio [aOR], 0.67; 95% CI, 0.59-0.76) and 42% lower risk in the left colon and rectum (aOR, 0.58; 95% CI, 0.48-0.71). There was no association with right colon cancers (aOR, 0.83; 95% CI, 0.69-1.01) but the difference in the estimates between the right colon and left colon or rectum was statistically significant (*P* = .01). FIT screening was associated with lower CRC mortality risk among non-Hispanic Asian (aOR, 0.37; 95% CI, 0.23-0.59), non-Hispanic Black (aOR, 0.58; 95% CI, 0.39-0.85) and non-Hispanic White individuals (aOR, 0.70; 95% CI, 0.57-0.86) (*P* for homogeneity = .04 for homogeneity).

**Conclusions and Relevance:**

In this nested case-control study, completing FIT was associated with a lower risk of overall death from CRC, particularly in the left colon, and the associations were observed across racial and ethnic groups. These findings support the use of FIT in population-based screening strategies.

## Key Points

**Question:**

What is the colorectal cancer mortality benefit of screening with fecal immunochemical tests (FITs)?

**Findings:**

In this nested case control study of 10 711 individuals, completing a FIT to screen for colorectal cancer was associated with a reduction in risk of dying from colorectal cancer of approximately 33% overall, and there was a 42% lower risk for left colon and rectum cancers. FIT screening was also associated with lower risk of colorectal cancer death among non-Hispanic Asian, non-Hispanic Black, and non-Hispanic White people.

**Meaning:**

This study provides US community-based evidence that suggests FIT screening lowers the risk of dying from colorectal cancer and supports the strategy of population-based screening using FIT.

## Teaser

This nested case-control study analyzed associations of fecal immunochemical test screenings with risk of death from colorectal cancer overall, by cancer location, and by race and ethnicity.

## Introduction

Colorectal cancer (CRC) is a major contributor to cancer deaths worldwide, and an estimated 152 810 individuals in the US received a diagnosis of CRC and 53 010 died from it in 2024.^1^ The US Preventive Services Task Force and other US organizations recommend annual fecal immunochemical test (FIT) screening among average-risk individuals to reduce the risk of death from CRC.^2,3^ FIT is simple to use, usually completed at home without the need for an in-person visit, and can be analyzed in a standardized way. FIT is more sensitive for both CRC and adenomas than guaiac-based fecal occult blood tests (g-FOBT) while being highly specific,^4,5,6,7^ and g-FOBT screening has also been shown to reduce the risk of CRC mortality.^4^

FIT screening programs have reported reduced CRC incidence and mortality,^8,9^ but further evidence on effectiveness is limited. Observational studies of biennial FIT screening in Europe and Taiwan have compared CRC mortality risk between screened and unscreened individuals or people invited vs not invited to screen in people aged 50 to 65 or 50 to 71 years.^10,11,12,13^ However, those studies did not verify eligibility in all individuals and/or used incidence-based mortality rates that is subject to lead-time bias. Current trials of FIT have limited power,^14^ and/or are not designed to compare FIT screening with unscreened individuals.^15^ This contrasts with observational and randomized clinical trial evidence on g-FOBT, sigmoidoscopy, and colonoscopy.^4,16,17,18,19^ Also, there are reasons to believe that FIT effectiveness may vary according to colon site^20,21,22^ and by race and ethnicity given differences in social and structural barriers that influence care quality across the screening continuum.^2,23^

We previously reported improved CRC incidence and mortality rates and narrowing of racial disparities in the Kaiser Permanente Northern California (KPNC) FIT-based screening program.^8,9^ This study examined whether completing FIT screening is associated with a lower risk of death from CRC overall, according to location in the colon, and by race and ethnicity. The approaches used in this study have previously generated findings that approximated randomized clinical trial results on sigmoidoscopy and g-FOBT.^24,25^

## Methods

### Study Design and Setting

This was a nested case-control study conducted among members of KPNC and KP Southern California (KPSC). The study followed the Strengthening the Reporting of Observational Studies in Epidemiology (STROBE) reporting guideline for case-control studies. KPNC and KPSC are multicenter, integrated, community-based health systems that provide both health insurance and clinical care to large, socially and geographically diverse populations. Both KPNC and KPSC implemented CRC screening programs starting in 2006 and 2007 that use proactive outreach with FIT (OC-AUTO FIT [Polymedco]) for all screening-eligible members who are not up to date by other means, such as colonoscopy. The health systems have standardized processes for delivering care across the screening continuum, from identifying eligible people to treatment for detected cancers, thusly facilitating the evaluation of screening test outcomes. All persons with a positive FIT are referred for follow-up colonoscopy and tracked to clinical resolution. Colonoscopy for primary screening is performed on request through referral to gastroenterology.^26,27^ The study was deemed exempt for review and the requirement of informed consent by the institutional review boards at The Ohio State University, KPNC and KPSC.

### Study Population and Sampling Approach

The study population included adults aged 52 to 85 years with at least 5 years of health plan membership prior to a reference date, which was the date of diagnosis for people who died of adenocarcinoma of the colon or rectum as the immediate or underlying cause between January 1, 2011, and December 31, 2017, as ascertained from tumor registry and state mortality files^25,28,29^ or a comparable reference date for selecting control persons. There was reasonable agreement in cause of death information among chart audits, registries, and the death certificate.^29^

The age criterion was based on screening guidelines during the study period, which recommended initiating screening at age 50 years and allowed 2 years (through age 52 years) for people to have opportunities to initiate screening. Extension to age 85 years was based on consideration of potential ongoing screening exposure and potential lagged (up to 5 years or longer) FIT screening mortality benefits.^2^ We used administrative codes in electronic databases to exclude people with a history of colectomy or increased-risk conditions for CRC, including gastrointestinal cancers, inflammatory bowel disease, inheritable genetic syndromes, or family history of CRC.^29,30^

Each case patient was individually matched using an incidence-density approach to 8 randomly selected people who were alive and not known to have CRC at the index date based on birth year (±1 year), sex, health plan membership duration prior to diagnosis (±1 year), and medical center and geographic region (Figure).^20,24^ The 1:8 matching ratio enhances statistical power. This approach enabled comparable periods of screening eligibility among case and control persons prior to the date of CRC diagnosis. To focus on the comparison of FIT screening exposure to those unscreened, we excluded people who had colonoscopy as the primary (or initiating) screening test during the 10-year period (Figure) but examined FIT to colonoscopy (485 participants) or sigmoidoscopy (51 participants) crossovers in sensitivity analyses. For more information on screening, see the eMethods in Supplement 1.

**Figure.**
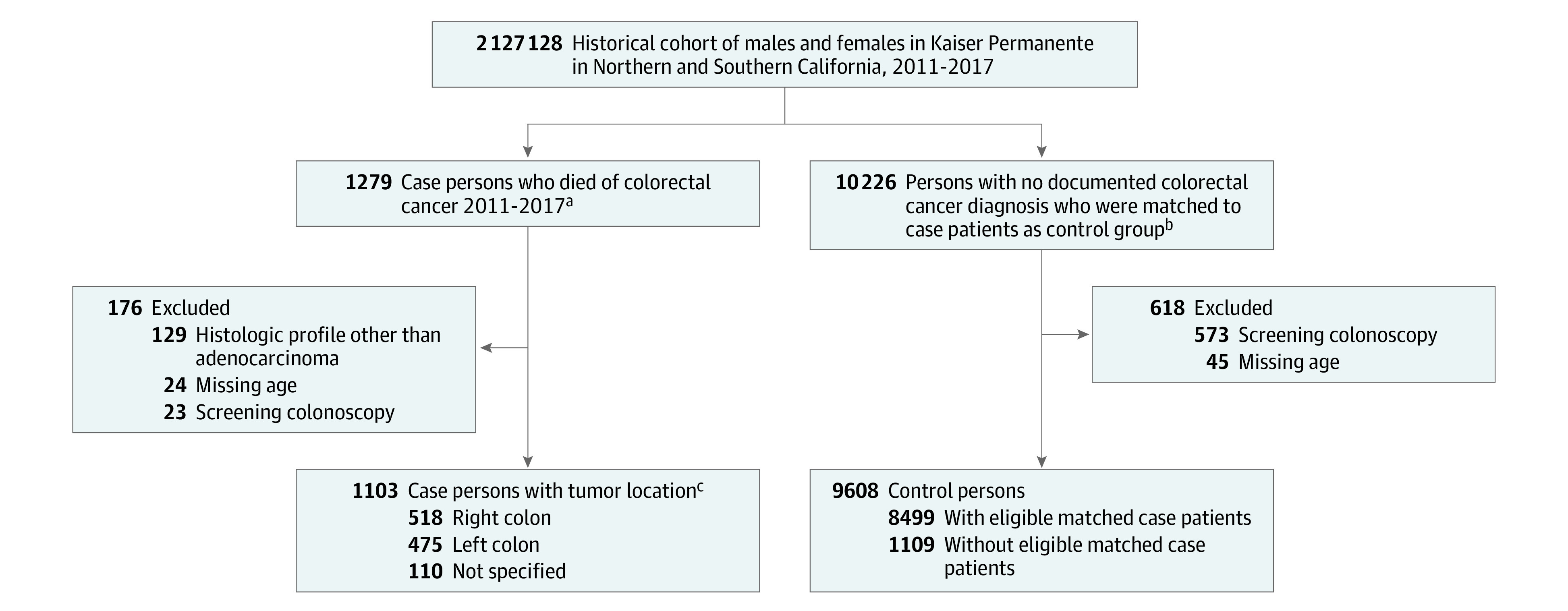
Flow of the Study Selection criteria at reference date included being aged 52 to 85 y during 2011 to 2017, 5 or more years prior enrollment in the health plan, and no prior gastrointestinal cancers, colectomy, inflammatory bowe disease, or genetic colorectal cancer syndromes in the 10 years prior to cohort entry. ^a^The date of colorectal cancer diagnosis was set as the reference and if unknown, the death date was used (32 participants). ^b^Matching was based on age, sex, health plan membership duration, and geographic region in a 1:8 ratio of case to control persons. Geographic regions were determined based on the medical center or facility in which a person received most of their care or were assigned for care by the health plan. ^c^The location was defined using the splenic flexure such that cancers that form in the colon proximal to the splenic flexure were classified as right colon cancers and those distal were left colon and rectum.

### Data Sources

Electronic databases that are derived from the electronic health record and administrative databases were used to obtain patients’ birth date, sex, race, ethnicity, health plan membership information, place of residence, and clinical information. Race and ethnicity data in the electronic health records are based on self-report (but may occasionally be assigned by an observer) and classified according to categories consistent with the US Office of Management and Budget 1997 Revisions to the Standards for the Classification of Federal Data on Race and Ethnicity. Consideration of race and ethnicity as a covariate was planned a priori*,* given differing social and economic experiences, colorectal adenoma prevalence in some studies, and CRC mortality risk.^2,23,31,32^ Socioeconomic status (SES) was assessed using data from the US Census Bureau at the tract level close to the midpoint of case ascertainment (2015).^33,34^ Clinical information included health care utilization, the clinician specialty and health care facilities visited, testing history, and diagnoses and procedures.^29^ Cancer diagnosis date, tumor location, and histologic examination findings were obtained from surveillance, epidemiology and end results program–affiliated tumor registries.^29^

### Colorectal Cancer Testing Data

Exposure to FIT was defined as completing 1 or more FITs with a valid laboratory result for screening prior to the reference date. FIT is primarily delivered by mail using systematic approaches. A positive result is based on a cutoff level of 20 or more μg of hemoglobin per gram of stool. We only included completed FITs documented as being performed in an outpatient setting. For each person included in the study, data on completion of FIT and other screening tests, including colonoscopy and cancer-related symptoms or signs, were ascertained during the 10-year period preceding the reference date. Electronic data were obtained on medical diagnoses, imaging studies, gastrointestinal endoscopies, and laboratory studies such as FIT.^25,28,29^ FITs preceded by colonoscopy and those performed in people with documented CRC-associated signs or symptoms were classified as nonscreening. We also applied a previously validated electronic algorithm as part of a multistep process to classify testing indication as diagnostic (eg, performed in people with iron deficiency anemia), surveillance, screening, or unknown.^28,29^ The algorithm used electronic data to assign indication as screening after excluding other indications. Thus, we selected people classified as receiving screening or surveillance colonoscopy along with a sample of people completing FIT for chart audit.^24,25,35^

### Covariates

We considered matching variables (including study site and geographic regions) as covariates. Race and ethnicity were categorized as non-Hispanic Asian, non-Hispanic Black, Hispanic or Latino, non-Hispanic White, or other (defined as Native American and Alaskan Native, multiracial and/or multiethnic, or unknown race and ethnicity). SES was measured using the percentage of people aged 25 years or older in a census tract without a high school diploma and categorized according to quartiles.^33,34^ Healthcare utilization was evaluated to assess health-seeking behavior by enumerating and categorizing (according to quartiles) the number of outpatient primary care clinician encounters in the 5 years prior to the reference date. The Charlson comorbidity score^36^ (0, 1, or ≥2) was used as a proxy of wellness to undergo screening. To evaluate whether the association of FIT screening with mortality varied across colon locations, we categorized cancers located proximal to the splenic flexure as right colon, those in the splenic flexure and distal locations as left colon and rectum, and others as not specified.

### Statistical Analysis

The primary analysis examined the association of completing FIT screening during the 5-year period prior (beginning from January 2007 through January 2013) to the reference date with the risk of CRC death during 2011 to 2017. In all analyses, people who did not receive any recommended CRC screening tests or received a CRC test for an indication other than screening during the relevant observation window served as the reference category. We also considered both shorter and longer intervals prior to the reference date during which FIT plausibly could identify preclinical cancer or precancer (ie, detectable preclinical period [DPP]).^20,25,37^ Because of the relatively high sensitivity of FIT for identifying advanced adenomas^4,5,6,7,38^ and because there is a greater threat to validity from underestimating than from overestimating the DPP duration,^39^ our analyses used a 5-year window as the primary approach.^20^

Analyses were performed overall (primary analysis) and by colon and rectum location. Secondary analyses were performed by race and ethnicity because protection from FIT depends on receiving follow-up colonoscopy for abnormal test result and social factors influence follow-up colonoscopy completion.^40^

In sensitivity analyses, we excluded people who had completed FIT screening before the relevant window due to the potential that people with prior negative FIT tests may represent a lower-risk population. Thus, in these additional analyses using the 5-year ascertainment window, we excluded people who completed FIT more than 5 years prior to the reference date. We also examined restrictions to people aged 55 years and older to maximize the available lookback period.

We evaluated both conditional and unconditional logistic regression analyses; given these yielded similar estimates, unconditional models were used to retain control persons for whom matched case persons were ineligible. The models were adjusted for matching variables (ie, age, sex, health plan membership duration, and geographic region), race and ethnicity, SES, comorbidity score, and wellness visits. All analyses were performed using the Stata statistical software version 18.0 (StataCorp). The threshold for significance was a 2-sided *P* < .05. Data analysis occurred from January 2002 to December 2017.

## Results

### Patient Characteristics

From an underlying population of 2 127 128 members during 2011 to 2017, we identified 1279 patients who died of CRC and 10 226 matched CRC-free persons (Figure). We excluded 129 case persons with a non-adenocarcinoma histologic profile and 24 case and 45 control persons with missing age information. We also excluded 23 case and 573 control persons who had only screening colonoscopy, resulting in a final study sample of 10 711 patients (3529 aged 60-69 years [32.9%]; 5587 male [52.1%] and 5124 female [47.8%]; 1254 non-Hispanic Asian [11.7%]; 973 non-Hispanic Black [9.1%]; 1929 Hispanic or Latino [18.0%]; 6345 non-Hispanic White [59.2%], 210 other race or ethnicity [2.0%]), including 1103 case and 9608 control patients, of whom 1109 did not have a matched case (Figure). Among those included in the analyses, there were fewer health care visits among case than control persons (Table 1).

**Table 1.** Characteristics of the Study Population, Kaiser Permanente Northern California and Kaiser Permanente Southern California (2011-2017)

Characteristics	Participants, No. (%)^a^		
	Case persons (n = 1103)	Control persons (n = 9608)	Total (N = 10 711)
Age, y^b^			
52-59	122 (11.1)	1103 (11.5)	1225 (11.4)
60-69	368 (33.4)	3161 (32.9)	3529 (32.9)
70-75	260 (23.6)	2223 (23.1)	2483 (23.2)
76-85	353 (32.0)	3121 (32.5)	3474 (32.4)
Sex			
Male	575 (52.1)	5012 (52.2)	5587 (52.2)
Female	528 (47.9)	4596 (47.8)	5124 (47.8)
Study site			
Kaiser Permanente Northern California	549 (49.8)	4706 (49)	5255 (49.1)
Kaiser Permanente Southern California	554 (50.2)	4902 (51)	5456 (50.9)
Membership duration, y			
5-10	215 (19.5)	1828 (19.0)	2043 (19.1)
11-15	562 (51.0)	4888 (50.9)	5450 (50.9)
16-20	326 (29.6)	2892 (30.1)	3218 (30.0)
Geographic region^c^			
1	72 (6.5)	598 (6.2)	670 (6.3)
2	162 (14.7)	1393 (14.5)	1555 (14.5)
3	73 (6.6)	636 (6.6)	709 (6.6)
4	62 (5.6)	510 (5.3)	572 (5.3)
5	63 (5.7)	555 (5.8)	618 (5.8)
6	117 (10.6)	1014 (10.6)	1131 (10.6)
7	81 (7.3)	748 (7.8)	829 (7.7)
8	120 (10.9)	1015 (10.6)	1135 (10.6)
9	130 (11.8)	1129 (11.8)	1259 (11.8)
10	83 (7.5)	776 (8.1)	859 (8.0)
11	83 (7.5)	721 (7.5)	804 (7.5)
12	57 (5.2)	513 (5.3)	570 (5.3)
Race and ethnicity^d^			
Non-Hispanic Asian	97 (8.8)	1157 (12)	1254 (11.7)
Non-Hispanic Black	141 (12.8)	832 (8.7)	973 (9.1)
Hispanic or Latino	190 (17.2)	1739 (18.1)	1929 (18.0)
Non-Hispanic White	671 (60.8)	5674 (59.1)	6345 (59.2)
Other or unknown^e^	4 (0.4)	206 (2.1)	210 (2.0)
Percentage of persons aged ≥25 y with <high school diploma in census tract, quartile			
1	258 (23.4)	2399 (25.0)	2657 (24.8)
2	258 (23.4)	2370 (24.7)	2628 (24.5)
3	289 (26.2)	2369 (24.7)	2658 (24.8)
4	290 (26.3)	2348 (24.4)	2638 (24.6)
Missing	8 (0.7)	122 (1.3)	130 (1.2)
Wellness visits 5 y prior to reference date, quartile^b^^,^^d^			
1	397 (36.0)	2401 (25.0)	2798 (26.1)
2	381 (34.5)	2847 (29.6)	3228 (30.1)
3	212 (19.2)	2229 (23.2)	2441 (22.8)
4	113 (10.2)	2131 (22.2)	2244 (21.0)
Charlson comorbidity index score^f^			
0	707 (64.1)	6222 (64.8)	6929 (64.7)
1	160 (14.5)	1554 (16.2)	1714 (16.0)
≥2	236 (21.4)	1832 (19.1)	2068 (19.3)

^a^ This table excludes 23 cases and 573 control persons who had screening colonoscopy during the 10-year period.

^b^ At the reference date, which is the date of diagnosis of colorectal adenocarcinoma; if unknown, the death date was used (n = 32).

^c^ Geographic regions were determined based on the medical center or facility in which a person received the majority of their care or are assigned for care by the health plan.

^d^ Pearson χ^2^ test of homogeneity *P* < .05.

^e^ Other or unknown includes Native American and Alaskan Native, multiracial and/or multiethnic as well as those with unknown race or ethnicity.

^f^ Ascertained during the 5-year period prior to the reference date.

During the 10-year period prior to the reference date, among control persons, 6101 (63.5%) completed at least 1 FIT screening, 4404 (45.8%) completed 2 or more FITs (eTable 1 in Supplement 1), and the FIT screening prevalence was relatively stable in 5 years preceding the reference date (eFigure 1 in Supplement 1). The cumulative FIT positive rate among control persons was 12.6% (768 controls), of whom 610 (79.4%) had colonoscopy within 12 months of the result date; the corresponding positivity and follow-up colonoscopy rates among control persons with ascertainment restricted to the 5-year period before the reference date were 10.6% (562 controls) and 76.8% (431 controls), respectively.

### Death From CRC and Mailed FIT Completion

During the 5 years prior to the reference date, 494 case persons (44.8%) and 5345 control persons (55.6%) completed 1 or more FIT screenings (Table 2). In unconditional logistic regression analyses, completing FIT screening was associated with a 33% lower risk of death from overall CRC (adjusted odds ratio [aOR], 0.67; 95% CI, 0.59-0.76) (Table 2).

**Table 2.** Completion of Mailed FIT and Risk of Death From Colorectal Cancer Overall and by Location

Location	Participants, No. (%) (N = 10 711)^a^		Adjusted OR (95% CI)^b^
	Case persons (n = 1103)	Control persons (n = 9608)	
Overall			
No screening	609 (55.2)	4263 (44.4)	1 [Reference]
FIT^c^	494 (44.8)	5345 (55.6)	0.67 (0.59-0.76)
Right colon^d^			
No screening	256 (49.4)	1804 (43.7)	1 [Reference]
FIT^c^	262 (50.6)	2323 (56.3)	0.83 (0.69-1.01)
Left colon or rectum^d^			
No screening	276 (58.1)	1615 (43.7)	1 [Reference]
FIT^c^	199 (41.9)	2081 (56.3)	0.58 (0.48-0.71)

Abbreviations: FIT, fecal immunochemical test; OR, odds ratio.

^a^ Excludes 23 cases and 573 controls who had screening colonoscopy during the 10-year period.

^b^ Logistic regression models adjusted for matching variables (ie, age, sex, health plan membership duration, and geographic region), race and ethnicity, socioeconomic status, comorbidity score, and wellness visits.

^c^ FIT use was defined as completion of one or more tests within 5 years of the reference date.

^d^ The difference in the estimates between the right colon and left colon or rectum was statistically significant (*P* = .01).

In stratified analyses, there was no statistically significant difference in CRC for right colon cancers (aOR, 0.83; 95% CI, 0.69-1.01), but there was a significant 42% lower risk of death for left colon and rectum cancers (aOR, 0.58; 95% CI, 0.48-0.71), and the difference in the estimates between the right colon and left colon or rectum was statistically significant (*P* = .01). In analyses stratified by race and ethnicity, completed FIT screenings were associated with a 63% lower risk of death for non-Hispanic Asian individuals (aOR, 0.37; 95% CI, 0.23-0.59), 42% lower risk among non-Hispanic Black individuals (aOR, 0.58; 95% CI, 0.39-0.85), and 29% lower risk among non-Hispanic White individuals (aOR, 0.71; 95% CI, 0.60-0.83). There was a 22% lower risk of death among Hispanic or Latino individuals, but this finding was not significant (aOR, 0.78; 95% CI, 0.57-1.08). There was statistically significant heterogeneity of the estimates across the groups (*P *for heterogeneity = .04) (Table 3).

**Table 3.** Screening FIT and Risk of Death From Colorectal Cancer Overall According to Race and Ethnicity

Race and ethnicity	Participants, No./Total No. (%)^a^		Adjusted OR (95% CI)^b^
	Case persons	Control persons	
Non-Hispanic Asian			
No screening	67/97 (69.1)	516/1157 (44.6)	1 [Reference]
FIT^c^^,^^d^	30/97 (30.9)	641/1157 (55.4)	0.37 (0.23-0.59)
Non-Hispanic Black			
No screening	84/141 (59.6)	407/832 (48.9)	1 [Reference]
FIT^c^^,^^d^	57/141 (40.4)	425/832 (51.1)	0.58 (0.39-0.85)
Hispanic or Latino			
No screening	96/190 (50.5)	754/1739 (43.4)	1 [Reference]
FIT^c^^,^^d^	94/190 (49.5)	985/1739 (56.6)	0.78 (0.57-1.08)
Non-Hispanic White			
No screening	360/671 (53.7)	2485/5674 (43.8)	1 [Reference]
FIT^c^^,^^d^	311/671 (46.3)	3189/5674 (56.2)	0.71 (0.60-0.83)

Abbreviations: FIT, fecal immunochemical test; OR, odds ratio.

^a^ The analyses excluded 23 cases and 573 controls who had screening colonoscopy during the 10-year period. Racial groups not shown had low sample sizes.

^b^ Logistic regression models adjusted for matching variables (ie, age, sex, health plan membership duration, geographic region), race and ethnicity, socioeconomic status, comorbidity score, and wellness visits.

^c^ FIT use was defined as completion within 5 years of the reference date.

^d^ The test of heterogeneity of the estimates was statistically significant (*P* = .04).

### Sensitivity Analysis

In analyses that excluded people with exposure to FIT prior to the 5-year period (892 cases and 7144 controls; eTable 2 in Supplement 1), FIT exposure was associated with a significant 31% lower risk of death from overall CRC (aOR, 0.69; 95% CI, 0.60-0.81) and left colon or rectum cancers (aOR, 0.69; 95% CI, 0.55-0.87). The difference in risk for right colon cancers was not significant (aOR, 0.81; 95% CI, 0.65-1.01) (eTable 3 in Supplement 1). Estimates stratified by race and ethnicity were similar to the unrestricted analysis (eTable 4 in Supplement 1).

Findings were stable to excluding crossovers to colonoscopy or sigmoidoscopy (aOR for overall CRC risk, 0.67; 95% CI, 0.58-0.76) and to excluding people younger than 55 years at death (eFigure 2 in Supplement 1). Sensitivity analyses with differing windows for FIT exposure ascertainment and differing age criteria produced similar results on overall CRC mortality as the primary analysis (eFigure 2 in Supplement 1). For instance, in analyses using the entire 10-year (rather than 5-year) observation period prior to the reference date, completing 1 or more FIT screenings was associated with a lower risk of death from overall CRC (aOR, 0.66; 95% CI, 0.58-0.75).

## Discussion

In this nested case-control study, we found that completing 1 or more FIT screenings within the prior 5 years was associated with a 33% lower risk of death from colorectal adenocarcinoma. The reduction in mortality risk was significant for those with left colon or rectum cancers (42%). The results are broadly similar to those obtained in randomized and nonrandomized studies of the association of g-FOBT with mortality from CRC and are consistent with observed reductions in CRC mortality rates following the initiation of organized screening.^8,9^ FIT has several practical advantages over g-FOBT for screening delivery, including improved adherence.^41^ Together with prior studies, the findings provide strong evidence for the long-term systematic delivery of FIT to reduce population rates of death from CRC, with evidence of benefit across the racial and ethnic groups examined.

The magnitude of the association differed according to tumor location within the colon and rectum, which is consistent with the results of prior CRC screening studies with both fecal testing and colonoscopy.^20,21,22,42,43^ A study by Selby and colleagues^21^ found that the mean stool hemoglobin concentration was 60.0 μg/g for left colon cancers and 12.4 μg/g for right colon cancers; thus, more cancers in the right colon than in the left colon would be expected to generate hemoglobin concentrations below the positivity threshold. It is possible that tumors in the right colon grow more rapidly, have higher frequency of being preceded by precursor lesions like sessile serrated polyps that are less likely to bleed and may be less detectable by FIT,^38,44^ or that the longer transit time leads to degradation of blood that is shed.^22^

This study was in 2 health care systems that have systematically delivered organized screening to a well-defined member population using population health management strategies for about 1.5 decades.^8,27,45^ FIT effectiveness in clinical practice depends upon receiving follow-up colonoscopy when the FIT result is abnormal. In the population studied, about 20% of people had not undergone a follow-up colonoscopy within 12 months of the result date. Although that follow-up rate is among the highest reported in the US,^46,47^ any failures to receive follow-up could diminish the potential effectiveness of FIT screening on reducing CRC mortality.

Our results are also similar to prior observational studies despite differences in settings, methods, populations, screening delivery (annual vs biennial), and age groups studied. Chiu et al^12^ found a lower risk of CRC death overall (adjusted rate ratio, 0.60; 95% CI, 0.57-0.64), for left colon cancers (adjusted rate ratio, 0.56; 95% CI, 0.53-0.69), and right colon cancers (adjusted rate ratio, 0.72; 95% CI, 0.66-0.80) after up to 10 years of follow-up in Taiwan’s national biennial screening program. The pooled analysis of g-FOBT trials reported a 25% reduction in CRC death among people who completed at least 1 screening round.^4^ Our estimated 33% lower risk may reflect that FIT has higher sensitivity and specificity. We leveraged the diversity of our population to report estimates that varied from 29% to 63% overall lower risk of CRC death in association with FIT screening across the racial and ethnic groups.

Well-designed case-control studies can produce valid estimates of the benefit of cancer screening in association with mortality but require being able to distinguish screening tests from those performed for work-up of CRC-related symptoms or signs. We used complementary approaches of electronic classification algorithms and medical record audits to assign test indication. Any residual misclassification would likely underestimate the true efficacy of FIT screening.^37^ Also, case-control studies of cancer screening and mortality require assumptions of the DPP. Our findings were largely unchanged in sensitivity analyses using varying FIT exposure ascertainment windows. Also, validity is enhanced if, during the time period corresponding to the DPP, screening prevalence is stable in the population from which the cases and controls are selected.^48^ With 1.5 decades of programmatic screening outreach, our study population had relatively stable screening prevalence during the FIT ascertainment window for our primary analyses and analyses using differing cutoff points and assumptions of the DPP yielded similar results.

### Limitations

This study has limitations. Almost one-half of people in our analyses had completed 2 or more FITs, but the case-control design is not suitable for assessing the impact of repeated screening (ie, strategy of annual FIT with perfect adherence) in part because positive screening results, which preclude further screening, are more common among case persons (eTable 1 in Supplement 1). In contrast, control persons without cancer are more likely to undergo repeated screening, leading to potentially spurious or exaggerated associations in analyses based on frequency of FIT screening. Although our findings may underestimate the effectiveness of FIT under conditions of perfect adherence, they reflect benefits likely to be observed in organized population-based screening but may not directly apply to populations with lower screening or follow-up colonoscopy adherence.

Our analysis accounted for potential confounders by exclusion, matching, stratification (eg, race and ethnicity and colon site), and model-based adjustment for socioeconomic and health care utilization history. However, the potential for confounding by healthy screenee effects remains. Our previous studies^49^ found that the likely magnitude of bias from residual confounding from unmeasured factors, such as lifestyle, is small and unlikely to change our findings. This study was conducted prior to the US Preventive Services Task Force recommendation to start screening at age 45 years^2^; thus, findings may not directly apply to people aged 45 to 49 years.

## Conclusions

In conclusion, this population-based nested case-control study observed that screening with 1 or more FIT was associated with a lower risk of dying from CRC, particularly for cancers in the left colon and rectum, with benefits observed across the racial and ethnic groups examined. The findings support the use of strategies for coordinated and equitable large-scale population-based delivery of FIT screening with follow-up of abnormal screening results to help avert preventable premature CRC deaths.

## Notes

Supplement 1. **eMethods.** Screening History **eTable 1.** Frequency of Fecal Immunochemical Test (FIT) Screening Among Case and Control Persons Over the 10-Year Ascertainment Window **eFigure 1.** Plot of Screening Prevalence by Years From Reference Date During the 10-Year Period Among Control Persons **eTable 2.** Characteristics of Study Population of People 52-85 Years Excluding People With FIT Exposure Prior to the Reference Date, KPNC/KPSC 2011-2017 **eTable 3.** Association Between Completion of Mailed FIT and Risk of Colorectal Cancer Death Overall and by Location Excluding People Who Received FIT Prior to the 5-Year Window **eTable 4.** Association Between Screening FIT and Risk of Death From Colorectal Cancer According to Race and Ethnicity, Excluding People With Prior FIT Exposure **eFigure 2.** Plot of the Association Between FIT Screening and Death From Colorectal Cancer Using Differing Lookback Periods and Inclusion Criteria Based on Age and Prior Screening

Supplement 2.
Data Sharing Statement
