# Supplementary material for: Fecal Immunochemical Test Screening and Risk of Colorectal Cancer Death
Source: JAMA Netw Open. 2024 Jul 19;7(7):e2423671. doi: 10.1001/jamanetworkopen.2024.23671 (PMC11259903; doi:10.1001/jamanetworkopen.2024.23671)
Supplement: Supplement 1. — eMethods. Screening History eTable 1. Frequency of Fecal Immunochemical Test (FIT) Screening Among Case and Control Persons Over the 10-Year Ascertainment Window eFigure 1. Plot of Screening Prevalence by Years From Reference Date During the 10-Year Period Among Control Persons eTable 2. Characteristics of Study Population of People 52-85 Years Excluding People With FIT Exposure Prior to the Reference Date, KPNC/KPSC 2011-2017 eTable 3. Association Between Completion of Mailed FIT and Risk of Colorectal Cancer Death Overall and by Location Excluding People Who Received FIT Prior to the 5-Year Window eTable 4. Association Between Screening FIT and Risk of Death From Colorectal Cancer According to Race and Ethnicity, Excluding People With Prior FIT Exposure eFigure 2. Plot of the Association Between FIT Screening and Death From Colorectal Cancer Using Differing Lookback Periods and Inclusion Criteria Based on Age and Prior Screening [file jamanetwopen-e2423671-s001.pdf]

Supplemental Online Content

Doubeni CA, Corley DA, Jensen CD, et al. Fecal immunochemical test screening and risk of colorectal cancer death. *JAMA Netw. Open*. 2024;7(7):e2423671. doi:10.1001/jamanetworkopen.2024.23671

eMethods. Screening History

eTable 1. Frequency of Fecal Immunochemical Test (FIT) Screening Among Case and Control Persons Over the 10-Year Ascertainment Window

eFigure 1. Plot of Screening Prevalence by Years From Reference Date During the 10-Year Period Among Control Persons

eTable 2. Characteristics of Study Population of People 52-85 Years Excluding People With FIT Exposure Prior to the Reference Date, KPNC/KPSC 2011-2017

eTable 3. Association Between Completion of Mailed FIT and Risk of Colorectal Cancer Death Overall and by Location Excluding People Who Received FIT Prior to the 5-Year Window

eTable 4. Association Between Screening FIT and Risk of Death From Colorectal Cancer According to Race and Ethnicity, Excluding People With Prior FIT Exposure

eFigure 2. Plot of the Association Between FIT Screening and Death From Colorectal Cancer Using Differing Lookback Periods and Inclusion Criteria Based on Age and Prior Screening

This supplemental material has been provided by the authors to give readers additional information about their work.

© 2024 Doubeni CA et al. *JAMA Network Open.*

eMethods. Screening History

Screening History

Screening history was determined in the study among control persons, which was drawn from the underlying population that resulted in the cases of colorectal adenocarcinoma during the 2011-2017 period. We excluded people who had received colonoscopy as their primary screening test. Prevalence of fecal immunochemical test (FIT) was determined by excluding tests performed within 10 years of a colonoscopy and tests classified as diagnostic or surveillance. We also excluded tests that were not documented as being performed in an outpatient location, as screening is typically completed at home and returned by mail for analysis.

Screening History among Control Persons

eFigure 1 shows the screening prevalence according to the year prior to the reference date demonstrating a higher prevalence when restricted to the 50-75-year-old age group for whom routine CRC screening is recommended by the US Preventive Services Task Force. Over the 10-year period of ascertainment, among control persons, the overall cumulative FIT screening prevalence (at least one screening FIT) was 63.5% (n=6,101). The cumulative FIT positive rate was 12.6% (n=768) among control persons. Overall, 68.6% (n=528), 75.7% (n=582) and 79.4% (n=610) of control persons with a positive FIT had follow-up colonoscopy within three, six, and 12 months, respectively.

When restricted to the 5-year period prior to the reference date, the FIT screening prevalence among control persons was 55.6% (n=5,345), of whom 10.6% (n=562) were positive. Among those with a positive FIT in the 5-year period, 65.5% (n=368), 73.4% (n=412), and 76.8% (n=431) had a follow-up colonoscopy performed within three, six, and 12 months, respectively.

© 2024 Doubeni CA et al. *JAMA Network Open.*

eTable 1 shows the distribution of the number of FITs received in the overall group and among people 50-75 years old. Among the control persons, about 45.8% had two or more FITs, and about 31.7% had 3 or more FITs. The proportions are a little less when restricted to 50-75-year-old age group.

Screening History among Case Persons

Among case persons, the overall FIT screening prevalence over the 10 years was 51.8% (n=283), 31.6% had two or more FITs and 19.4% had three or more FITs (***eTable 1***). The cumulative FIT positive rate was 49.7% among case persons.

When restricted to the 5-year period prior to the reference date, the screening prevalence among case persons was 44.8% (n=494), of whom 51.3% were positive.

© 2024 Doubeni CA et al. *JAMA Network Open.*

eTable 1. Frequency of Fecal Immunochemical Test (FIT) Screening Among Case and Control Persons Over the 10-Year Ascertainment Window

Number of FIT completed, n(%)	Case persons (N=1,103)	Control persons (N=9,608)
**Entire sample, overall***		
0	532 (48.2)	3507 (36.5)
1	223 (20.2)	1697 (17.7)
2	135 (12.2)	1357 (14.1)
3	87 (7.9)	1056 (11)
4	57 (5.2)	818 (8.5)
5+	69 (6.3)	1173 (12.2)
**People 50-75, overall***		
0	572 (51.9)	3810 (39.7)
1	210 (19.0)	1748 (18.2)
2	128 (11.6)	1255 (13.1)
3	81 (7.3)	957 (10.0)
4	53 (4.8)	758 (7.9)
5+	59 (5.3)	1080 (11.2)

© 2024 Doubeni CA et al. *JAMA Network Open.*

eFigure 1. Plot of Screening Prevalence by Years From Reference Date During the 10-Year Period Among Control Persons

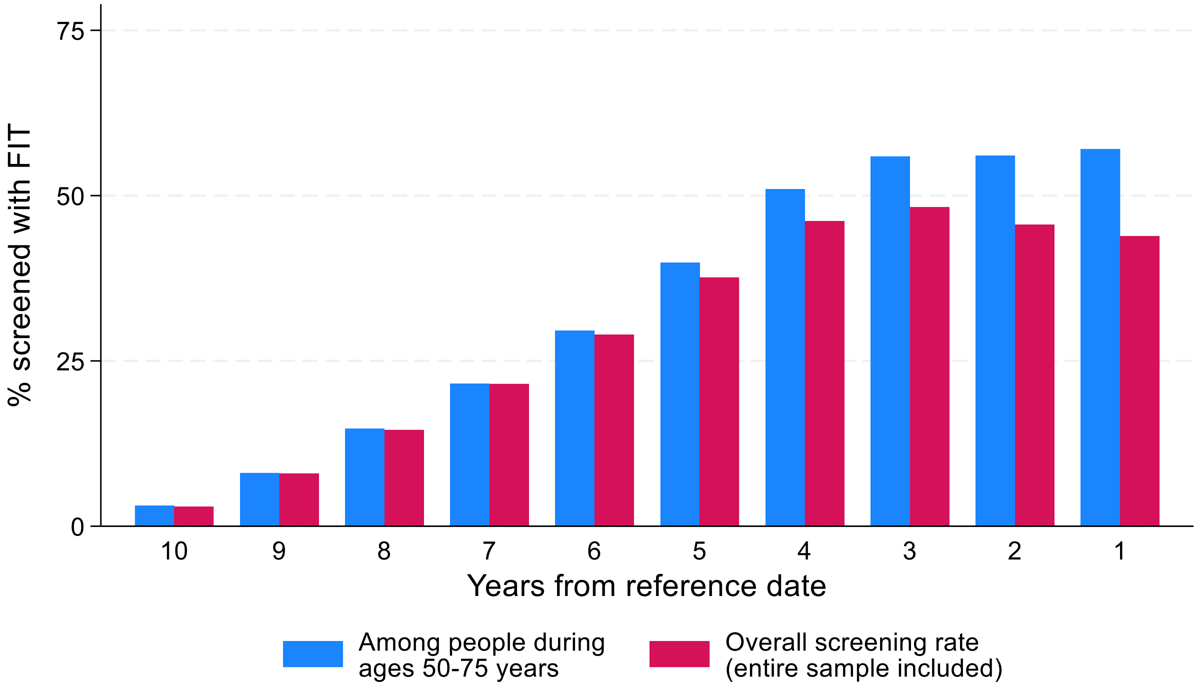

© 2024 Doubeni CA et al. *JAMA Network Open.*

eTable 2. Characteristics of Study Population of People 52-85 years Excluding People With FIT Exposure Prior to the Reference Date, KPNC/KPSC 2011-2017

**Characteristics, n (%)**	**Case persons (N=892)**	**Control persons (N=7,144)**	**Total (N=8,036)**
**Age, years** (at reference date*)			
52-59	112 (12.6)	972 (13.6)	1,084 (13.5)
60-69	313 (35.1)	2,428 (34.0)	2,741 (34.1)
70-75	213 (23.9)	1,708 (23.9)	1,921 (23.9)
76-85	254 (28.5)	2,036 (28.5)	2,290 (28.5)
**Female**	422 (47.3)	3,372 (47.2)	3,794 (47.2)
**Study site**			
KPNC	444 (49.8)	3,409 (47.7)	3,853 (47.9)
KPSC	448 (50.2)	3,735 (52.3)	4,183 (52.1)
**Membership duration, years**			
5-10	191 (21.4)	1475 (20.6)	1,666 (20.7)
11-15	494 (55.4)	4186 (58.6)	4,680 (58.2)
16-20	207 (23.2)	1483 (20.8)	1,690 (21.0)
**Geographic location**†			
1	60 (6.7)	411 (5.8)	471 (5.9)
2	133 (14.9)	1,010 (14.1)	1,143 (14.2)
3	55 (6.2)	458 (6.4)	513 (6.4)
4	51 (5.7)	402 (5.6)	453 (5.6)
5	47 (5.3)	356 (5.0)	403 (5.0)

© 2024 Doubeni CA et al. *JAMA Network Open.*

6	98 (11.0)	772 (10.8)	870 (10.8)
7	65 (7.3)	617 (8.6)	682 (8.5)
8	101 (11.3)	802 (11.2)	903 (11.2)
9	98 (11.0)	839 (11.7)	937 (11.7)
10	63 (7.1)	541 (7.6)	604 (7.5)
11	70 (7.8)	559 (7.8)	629 (7.8)
12	51 (5.7)	377 (5.3)	428 (5.3)
**Race and ethnicity**§			
Non-Hispanic White	83 (9.3)	864 (12.1)	947 (11.8)
Non-Hispanic Black	118 (13.2)	671 (9.4)	789 (9.8)
Non-Hispanic Asian	161 (18.0)	1,274 (17.8)	1,435 (17.9)
Hispanic/Latino	528 (59.2)	4,177 (58.5)	4,705 (58.5)
Other or Unknown	2 (0.2)	158 (2.2)	160 (2.0)
**Number of persons 25+ years in census tract < high school diploma (Quartiles)**			
Q1	204 (22.9)	1,820 (25.5)	2,024 (25.2)
Q2	205 (23.0)	1,710 (23.9)	1,915 (23.8)
Q3	239 (26.8)	1,759 (24.6)	1,998 (24.9)
Q4	236 (26.5)	1,760 (24.6)	1,996 (24.8)
Missing	8 (0.9)	95 (1.3)	103 (1.3)
**Wellness visits 5 years prior to reference date***§			
Q1	346 (38.8)	1,854 (26.0)	2,200 (27.4)
Q2	314 (35.2)	2,152 (30.1)	2,466 (30.7)
Q3	161 (18.0)	1,636 (22.9)	1,797 (22.4)
Q4	71 (8.0)	1,502 (21.0)	1,573 (19.6)
**Charlson score‡**			

0	598 (67.0)	4,701 (65.8)	5,299 (65.9)
1	124 (13.9)	1,157 (16.2)	1,281 (15.9)
≥2	170 (19.1)	1,286 (18.0)	1,456 (18.1)

Note: this table also excludes 23 case and 573 control persons who had screening colonoscopy during the 10-year period.

* The reference date is the date of diagnosis of colorectal adenocarcinoma and if unknown, the date of death from colorectal adenocarcinoma.

** Other or Unknown includes Native American and Alaskan Native, Multiracial and/or Multiethnic as well as those with unknown racial and/or ethnic group assignment.

† Geographic regions were determined based on the medical center in which they received most of their care or are assigned for care by the health plan.

‡Ascertained during the 5-year period prior to the reference date.

§Pearson Chi-Square for test homogeneity yield a p-value of <0.05

KPNC. Kaiser Permanente Northern California; KPSC, Kaiser Permanente Southern California; Q, quartile.

© 2024 Doubeni CA et al. *JAMA Network Open.*

eTable 3. Association Between Completion of Mailed FIT and Risk of Colorectal Cancer Death Overall and by Location Excluding People Who Received FIT Prior to the 5-Year Window*

Overall and colon site	**Case persons[†] n (%)**	**Control persons n (%)**	**Adjusted OR (95% CI)[††]**
**Overall**			
No Screening	540 (60.5)	3,604 (50.5)	Ref
FIT**	352 (44.9)	3,540 (49.5)	0.69 (0.60-0.81)
**Right Colon**			
No Screening	224 (56.1)	1,552 (49.9)	Ref
FIT**	175 (43.9)	1,557 (50.1)	0.81 (0.65-1.01)
**Left Colon/rectum**			
No Screening	242 (61.1)	1,371 (49.8)	Ref
FIT**	154 (38.9)	1,385 (50.2)	0.69 (0.55-0.87)

*Comprised of people 52-85 years and excluded those who had FIT >5 years prior to the reference date

†Analyses stratified on left and right colon/rectum excludes 97 case persons with unspecified tumor location

††Logistic regression models adjusted for matching variables (i.e., age, sex, health plan enrollment duration, geographic region), race and ethnicity, socioeconomic status, comorbidity score, and wellness visits

**FIT (fecal immunochemical test) use defined as completion within the 5-year period prior to the reference date.

OR, odds ratio; CI, confidence interval.

© 2024 Doubeni CA et al. *JAMA Network Open.*

eTable 4. Association Between Screening FIT and Risk of Death From Colorectal Cancer According to Race and Ethnicity, Excluding People With Prior FIT Exposure†

	**Case persons n (%)**	**Control persons n (%)**	**Adjusted OR (95% CI) ††**
**Non-Hispanic Asian**			
No Screening	59 (71.1)	437 (50.6)	Ref
FIT*	24 (28.9)	427 (49.4)	0.46 (0.27-0.78)
**Non-Hispanic Black**			
No Screening	78 (66.1)	370 (55.1)	Ref
FIT*	40 (33.9)	301 (44.9)	0.55 (0.35-0.87)
**Hispanic or Latino**			
No Screening	88 (54.7)	633 (49.7)	Ref
FIT*	73 (45.3)	641 (50.3)	0.87 (0.61-1.24)
**Non-Hispanic White**			
No Screening	314 (59.5)	2,076 (49.7)	Ref
FIT*	214 (40.5)	2,101 (50.3)	0.71 (0.59-0.87)

†Excludes people with exposure to FIT >5 years prior to the reference date. Some racial groups are not shown due to small sample sizes.

††Logistic regression models adjusted for matching variables (i.e., age, sex, health plan enrollment duration, geographic region), race and ethnicity, socioeconomic status, comorbidity score, and wellness visits

*FIT (fecal immunochemical test) use defined as completion <5 years of the reference date.

OR, odds ratio; CI, confidence interval.

© 2024 Doubeni CA et al. *JAMA Network Open.*

eFigure 2. Plot of the Association Between FIT Screening and Death From Colorectal Cancer Using Differing Lookback Periods and Inclusion Criteria Based on Age and Prior Screening

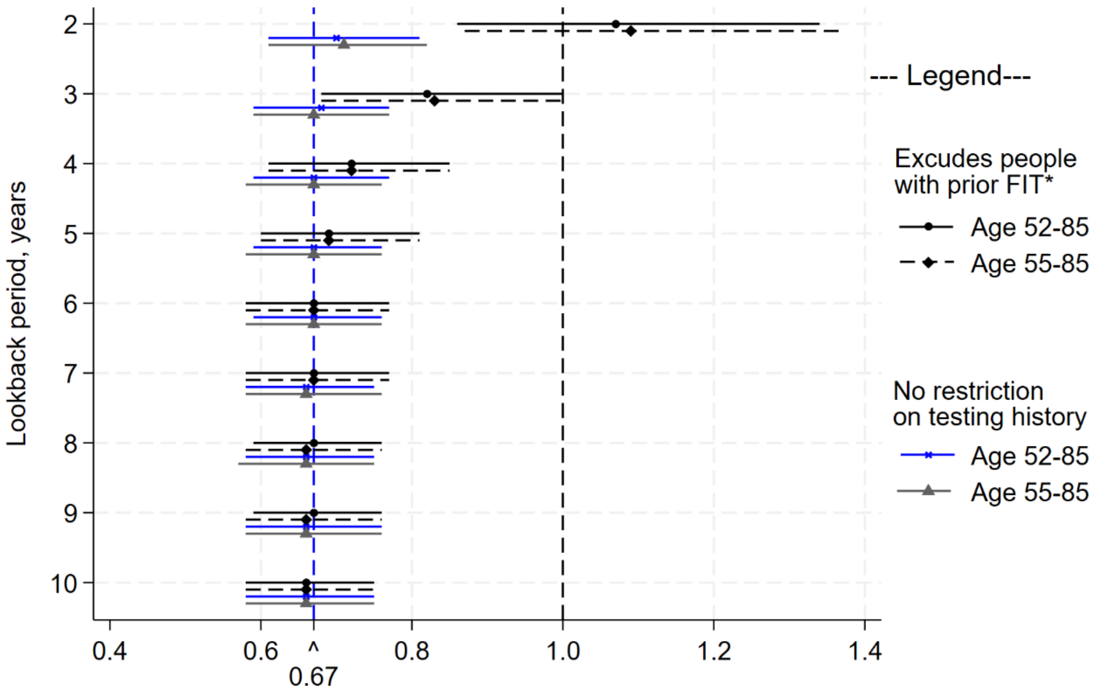

^ Point estimate (0.67) from the primary analysis

*These groups exclude people who completed FIT prior to the relevant lookback or screening ascertainment window. For instance, the 2-year window (y-axis) excluded people who had received FIT in the 3-10 years prior to the reference, which results in a biased estimate as demonstrated.

FIT, fecal immunochemical test; CI, confidence interval.

© 2024 Doubeni CA et al. *JAMA Network Open.*
