# Supplementary material for: Fecal Immunochemical Test Screening and Risk of Colorectal Cancer Death
Source: JAMA Netw Open. 2024 Jul 19;7(7):e2423671. doi: 10.1001/jamanetworkopen.2024.23671 (PMC11259903; doi:10.1001/jamanetworkopen.2024.23671)
Supplement: Supplement 2. — Data Sharing Statement [file jamanetwopen-e2423671-s002.pdf]

# Data Sharing Statement

Doubeni. Fecal Immunochemical Test Screening and Risk of Colorectal Cancer Death. *JAMA Netw Open*. Published July 19, 2024. doi:10.1001/jamanetworkopen.2024.23671

**Data**
**Data available:** Yes
**Data types:** Deidentified participant data
**How to access data:** Contact at Chyke.Doubeni@osumc.edu
**When available:** With publication

**Supporting Documents**
**Document types:** Statistical/analytic code
**How to access documents:** Contact at Chyke.Doubeni@osumc.edu
**When available:** With publication

**Additional Information**
**Who can access the data:** researchers with approved proposed use
**Types of analyses:** Replication of findings
**Mechanisms of data availability:** with data access agreement
